# Meta-analysis of differentially expressed genes in osteosarcoma based on gene expression data

**DOI:** 10.1186/1471-2350-15-80

**Published:** 2014-07-14

**Authors:** Zuozhang Yang, Yongbin Chen, Yu Fu, Yihao Yang, Ya Zhang, Yanjin Chen, Dongqi Li

**Affiliations:** 1 Bone and Soft Tissue Tumors Research Center of Yunnan Province, Department of Orthopaedics, The Third Affiliated Hospital of Kunming Medical University (Tumor Hospital of Yunnan Province), Kunming, Yunnan 650118, PR China; 2 Key Laboratory of Animal Models and Human Disease Mechanisms, Kunming Institute of Zoology, Chinese Academy of Sciences, Kunming, Yunnan 650223, PR China; 3 Department of Dermatology, Beijing Hospital, Beijing 100730, PR China

**Keywords:** Osteosarcoma, Expression data, Meta-analysis, Microarray, Differentially expressed genes

## Abstract

**Background:**

To uncover the genes involved in the development of osteosarcoma (OS), we performed a meta-analysis of OS microarray data to identify differentially expressed genes (DEGs) and biological functions associated with gene expression changes between OS and normal control (NC) tissues.

**Methods:**

We used publicly available GEO datasets of OS to perform a meta-analysis. We performed Gene Ontology (GO) enrichment analysis, Kyoto Encyclopedia of Genes and Genomes (KEGG) pathway analysis and Protein-Protein interaction (PPI) networks analysis.

**Results:**

Eight GEO datasets, including 240 samples of OS and 35 samples of controls, were available for the meta-analysis. We identified 979 DEGs across the studies between OS and NC tissues (472 up-regulated and 507 down-regulated). We found GO terms for molecular functions significantly enriched in protein binding (GO: 0005515, P = 3.83E-60) and calcium ion binding (GO: 0005509, P = 3.79E-13), while for biological processes, the enriched GO terms were cell adhesion (GO:0007155, P = 2.26E-19) and negative regulation of apoptotic process (GO: 0043066, P = 3.24E-15), and for cellular component, the enriched GO terms were cytoplasm (GO: 0005737, P = 9.18E-63) and extracellular region (GO: 0005576, P = 2.28E-47). The most significant pathway in our KEGG analysis was Focal adhesion (P = 5.70E-15). Furthermore, ECM-receptor interaction (P = 1.27E-13) and Cell cycle (P = 4.53E-11) are found to be highly enriched. PPI network analysis indicated that the significant hub proteins containing PTBP2 (Degree = 33), RGS4 (Degree = 15) and FXYD6 (Degree = 13).

**Conclusions:**

Our meta-analysis detected DEGs and biological functions associated with gene expression changes between OS and NC tissues, guiding further identification and treatment for OS.

## Background

Osteosarcoma (OS), the most common non-haematological primary malignant tumor of bone, occurs most commonly in the metaphyseal regions of long bones mainly in adolescents and young adults, but also in patients over 40 years of age [1]. Though the survival rate has been improved after the introduction of neoadjuvant chemotherapy, the need for advances in treatments is still very urgent [2,3]. Therefore, an in-depth understanding of the pathobiology of OS is needed to develop rational treatment options for OS. Cytogenetic analyses have revealed that most conventional OShave complex karyotypes with numerous and highly variable genomic aberrations [4]. Many genes become dysregulated due to genomic aberrations, and DNA copy number and DNA methylationand and gene expression data combined to identify oncogenes and tumor suppressor genes in OS [5,6].

As the high-throughput technologies have been used in many fields, detection of expression level across the whole genome is a useful way to find unusual genomic alteration in OS patients with microarray. Recently, researchers have used this technique to more comprehensively increase knowledge about the cellular and molecular changes in OS [7-13]. Although these studies have shown lists of differently expressed genes (DEGs), there tends to be inconsistencies among studies due to limitations of small sample sizes and varying results obtained by different groups, accomplished by different laboratory protocols, microarray platforms and analysis techniques [14]. Recent studies have shown that the systematic integration of gene expression data from multiple sources, so-called meta-analyses, can increase statistical power for detecting differentially expressed genes while allowing for an assessment of heterogeneity, and may lead to more robust, reproducible and accurate predictions [15,16]. Similar meta-analysis has never been conducted for OS, and we first perform a meta-analysis of gene expression data sets from various OS studies to overcome the limitations of individual studies, resolve inconsistencies, and reduce the likelihood that random errors are responsible for false-positive or false-negative associations, and lay a foundation for uncovering the pathology of OS and further generating new therapies for OS.

## Methods

### Identification of eligible OS gene expression datasets

**Figure 1:**
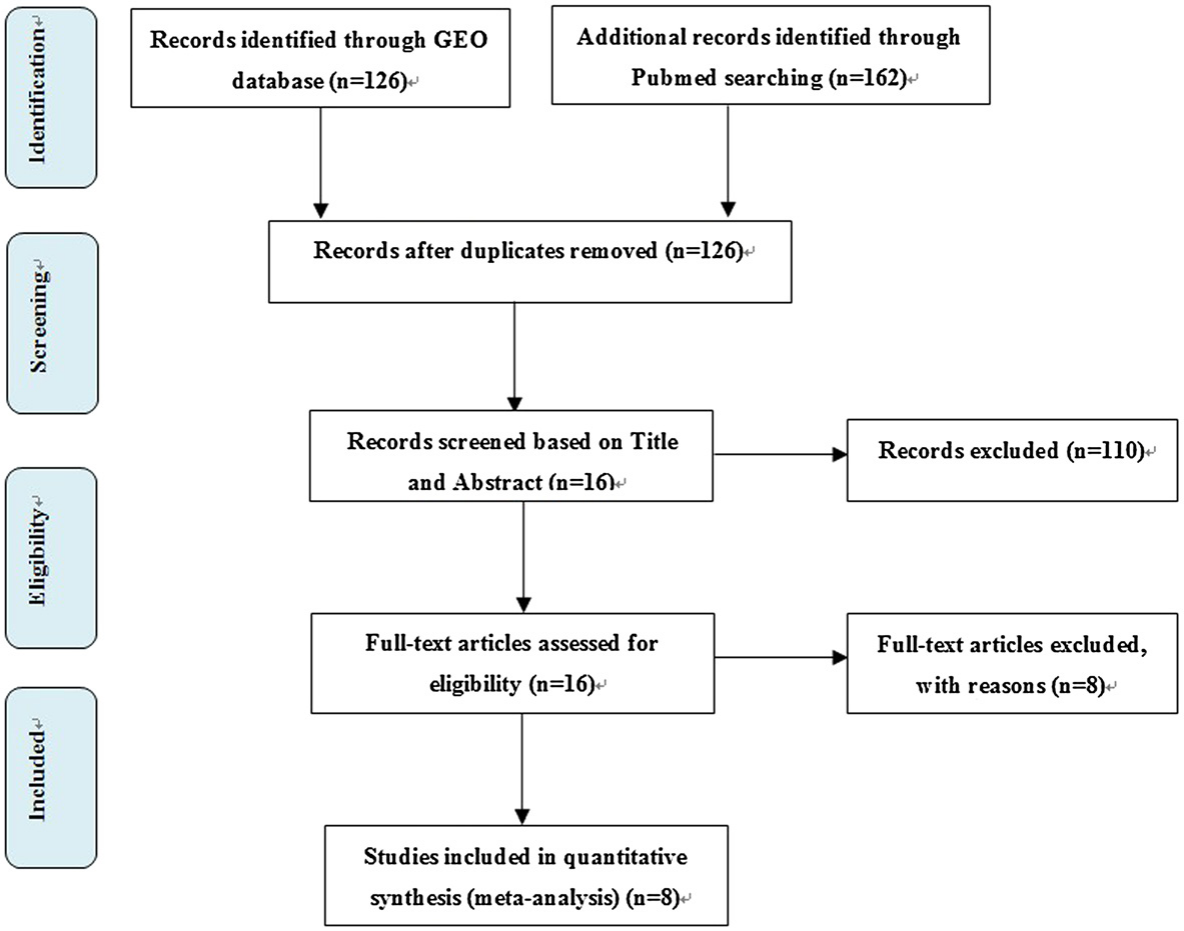
Flowchart of the selected process of microarray datasets for the meta-analysis.

OS expression profiling studies were identified by searching PubMed database. The following key words and their combinations were used: “osteosarcoma, gene expression, microarray, genetics”. In addition, the Gene Expression Omnibus database (GEO, http://www.ncbi.nlm.nih.gov/geo) was also searched to ensure the relevant studies were not missed [17]. We only retained the original experimental articles that analyzed gene expression profiling between OS and normal control (NC) tissues. Non-human studies, review articles and integrated analysis of expression profiles were excluded (Figure 1). We conducted this meta-analysis in accordance with the guidelines provided in the PRISMA statement (Additional file 1: The PRISMA Checklist S1). Data were extracted from the original studies by 2 independent reviewers. Any discrepancies between reviewers were resolved by consensus or a third reviewer. The following information was extracted from each identified study: GEO accession number, sample type, platform, number of cases and controls, references, and gene expression data.

### Data preprocessing

Normalization is important for comparison of microarray data sets. The heterogeneity of different datasets caused by different platforms, different gene nomenclature and different control tissues may make it difficult to directly compare the data sets from various sources. The improperly normalization used in the comparisons of microarray data sets may run a high risk of skewing comparison results and reduce the credibility of measurements of individual gene expression change. In this case, a global normalization method to minimize the inconsistency should be considered. For this purpose, we used the Z-score transformation approach to calculate the expression intensities of each probe in gene expression profiles. Z-scores were calculated according to the following formula:

$$Z\;\mathrm{score}=\frac{x_{i}‒\bar{x}}{\delta }$$

Where x_i_ represents raw intensity data for each gene; $\bar{x}$represents average gene intensity within a single experiment and δ represents standard deviation (SD) of all measured intensities.

### Statistical analysis

The significance analysis of microarray (SAM) software was then used to identify the DEGs between pathological and control samples. This procedure identifies DEGs by carrying out gene specific t-statistics, with a “relative difference” score for each gene. The D value was defined as the average expression change from different expression states to the standard deviation of measurements for that gene. Genes exhibiting at least two-fold changes corresponding to a false discovery rate (FDR) less than 0.05 were selected as the significantly DEGs [18].

### Functional classification of DEGs

In order to interpret the biological significance of the DEGs, we performed Gene Ontology (GO) enrichment analysis to investigate their functional distribution in OS. The online based software GENECODIS (http://genecodis.cnb.csic.es) was used to perform this analysis [19]. In addition, we also performed the pathway enrichment analysis based on the Kyoto Encyclopedia of Genes and Genomes (KEGG) database.

### PPI network construction

The protein-protein interactions (PPIs) research could reveal the functions of proteins at the molecular level and help discover the rules of cellular activities including growth, development, metabolism, differentiation and apoptosis [20]. The identification of protein interact ions in a genome-wide scale is an important step for the interpretation of the cellular control mechanisms [21]. In this analysis, we used Biological General Repository for Interaction Datasets (BioGRID) (http://thebiogrid.org/) to construct PPI network and visualized the distribution characteristics of the top 10 up- and down-regulated DEGs in the network in Cytoscape [22].

## Results

### Short overview of the studies included

In this work, we collected a total of 8 expression profiling studies according to the inclusion criteria, among which it included240 samples of OS and 35 samples of controls. Selected details of the individual studies are summarized in Table 1. 4 studies utilized bone tissues of OS samples, 3 studies utilized OS cell lines and 1 study utilized bone tissues and cell lines simultaneously.

**Table 1:** Characteristics of the individual studies

**GEO ID**	**Sample count (case:control)**	**Platform**	**Sample source**	**Tissue**
GSE14359	18: 2	GPL96 [HG-U133A] Affymetrix Human Genome U133A Array	in vivo	Bone, lung
GSE16102	48: 6	GPL96 [HG-U133A] Affymetrix Human Genome U133A Array	in vivo	Bone
GSE12865	12: 2	GPL6244 [HuGene-1_0-st] Affymetrix Human Gene 1.0 ST Array	in vivo	Bone
GSE11414	4: 2	GPL6244 [HuGene-1_0-st] Affymetrix Human Gene 1.0 ST Array	in vitro	Bone
GSE42352	103:15	GPL10295 Illumina human-6 v2.0 expression beadchip (using nuIDs as identifier)	in vivo/in vitro	Bone
GSE36001	19: 6	GPL6102 Illumina human-6 v2.0 expression beadchip	in vitro	Bone
GSE32964	35: 1	GPL6947 Illumina HumanHT-12 V3.0 expression beadchip	in vivo	Bone
GSE30807	1: 1	GPL570 [HG-U133_Plus_2] Affymetrix Human Genome U133 Plus 2.0 Array	in vitro	Bone

### Detecting genes associated with OS

To identify the genetic markers involved in the development and progression of OS, we firstly unified the probe ID, for a microarray platform that represent a named gene, and the HUGO symbol of that gene, to the Entrez gene ID. The expression value was logarithmically transformed (base 2). This lead to a total of 14722 genes obtained. The expression values for each gene were then transformed to the z-score for the purpose of global normalization. By using the assembled expression compendium, we investigated the global shifts of gene expression between OS and NC. Then, we used SAM method to identify DEGs between pathological and control samples. With a FDR of 0.05 and by applying a minimal fold change of 1.4, a total of 979 genes were found to show altered expression in samples of OS compared with normal control. Among those DEGs, 472 genes were up-regulated and 507 genes were down-regulated. A list of the top 10 most significantly up- or down-regulated DEGs was presented in Table 2. The up-regulated gene with the lowest P-value (P = 5.08E-15) was CPE (carboxypeptidase E), previously reported to correlate with tumor growth and metastasis [23], which is a carboxypeptidase that cleaves C-terminal amino acid residues and is involved in the biosynthesis of peptide hormones and neurotransmitters, including insulin [24]. The down-regulated gene with the lowest P-value (P = 1.86E-48) was NPR3 (natriuretic peptide receptor 3) that acts as a decoy/clearance receptor functioning to limit the effects of natriuretic peptides. The full list of these genes was provided as Additional file 2: Table S1.

**Table 2:** The top 10 most significantly up- or down-regulated DEGs

**Gene ID**	**Gene symbol**	**P-value**	**Fold change**	**Official full name**
**Up-regulated genes**				
1363	CPE	5.08E-15	3.856	Carboxypeptidase E
23462	HEY1	6.72E-15	2.7097	Hes-related family bHLH transcription factor with YRPW motif 1
5538	PPT1	8.34E-15	2.7668	Palmitoyl-protein thioesterase 1
58155	PTBP2	2.19E-12	1.7278	Polypyrimidine tract binding protein 2
81035	COLEC12	3.87E-12	1.7098	Collectin sub-family member 12
54504	CPVL	4.90E-12	2.4601	Carboxypeptidase, vitellogenic-like
53826	FXYD6	2.40E-11	2.3862	FXYD domain containing ion transport regulator 6
54453	RIN2	5.85E-11	1.6714	Ras and Rab interactor 2
80135	RPF1	5.94E-11	1.7889	Ribosome production factor 1 homolog (S. cerevisiae)
1942	EFNA1	1.23E-10	1.9501	Ephrin-A1
**Down-regulated genes**				
4883	NPR3	1.9E-48	−2.352	Natriuretic peptide receptor 3
25802	LMOD1	4.1E-32	−1.5799	Leiomodin 1 (smooth muscle)
2621	GAS6	2.5E-31	−2.6934	Growth arrest-specific 6
5959	RDH5	4.2E-30	−2.3838	Retinol dehydrogenase 5 (11-cis/9-cis)
316	AOX1	3.2E-28	−2.1826	Aldehyde oxidase 1
55679	LIMS2	9.2E-28	−1.7498	LIM and senescent cell antigen-like domains 2
4053	LTBP2	7.3E-27	−3.0523	Latent transforming growth factor beta binding protein 2
5999	RGS4	8.3E-27	−3.2551	Regulator of G-protein signaling 4
8613	PPAP2B	2.2E-25	−2.6475	Phosphatidic acid phosphatase type 2B
23529	CLCF1	6.5E-25	−1.6977	Cardiotrophin-like cytokine factor 1

### Functional annotation

**Figure 2:**
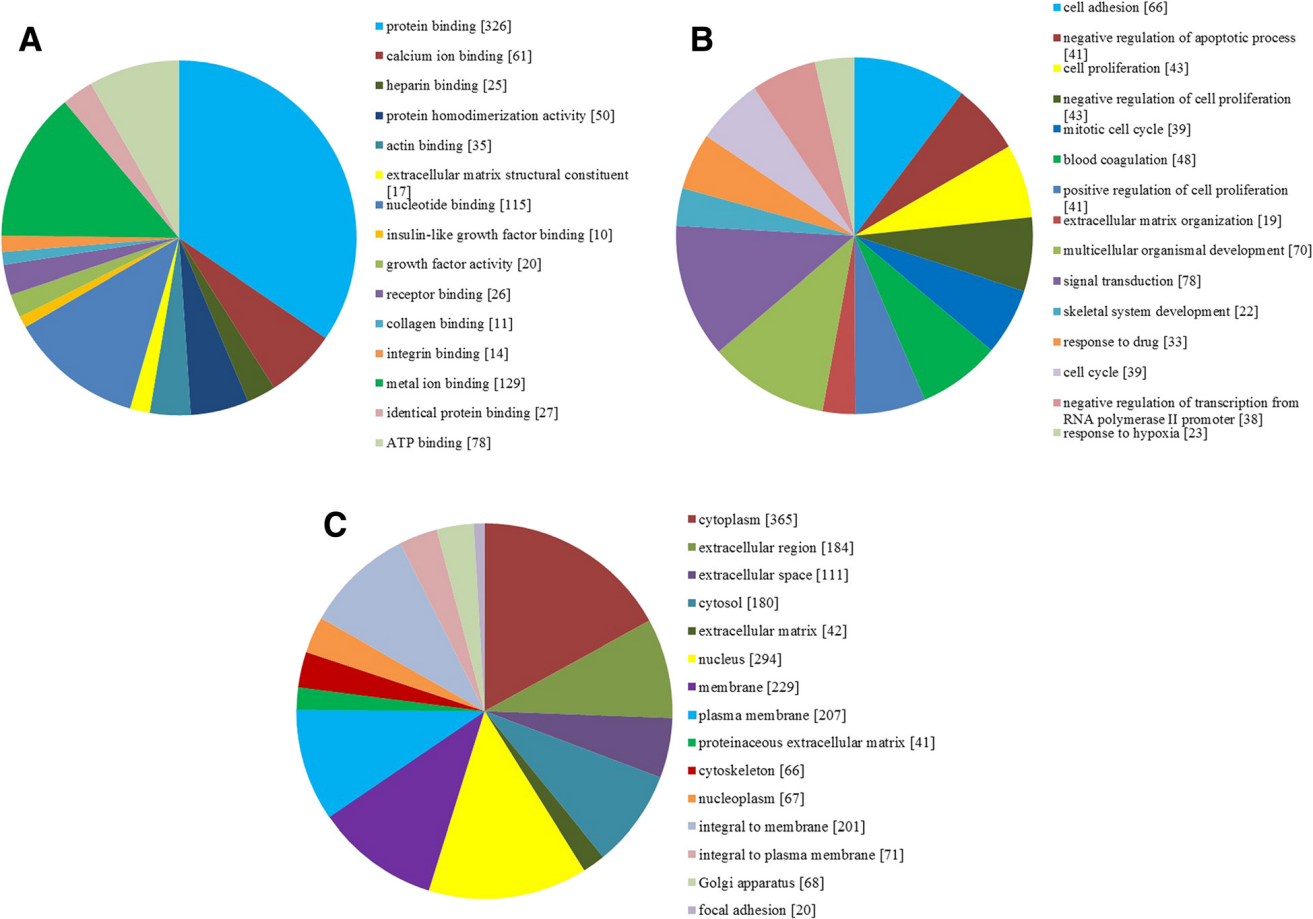
**The top 15 enriched GO terms of differentially expressed genes. A**. molecular functions for DEGs (P value ≤ 9.35E-06); **B**. biological process for DEGs (P value ≤ 1.92E-07); **C**. cellular component for DEGs (P value ≤ 2.98E-09).

To gain insights into the biological roles of the DEGs from OS, we performed a GO categories enrichment analysis. Gene ontology provides a common descriptive framework and functional annotation and classification for analyze the gene sets data. GO categories are organized into three groups: biological process, cellular component, and molecular function. The biological process and molecular functions are thus examined separately in our analysis by web-based software GENECODIS. Genes that showed a nominal significance level of P < 0.01 were selected and were tested against the background set of all genes with GO annotations. We found GO terms for molecular functions significantly enriched in protein binding (GO: 0005515, P = 3.83E-60) and calcium ion binding (GO: 0005509, P = 3.79E-13), while for biological processes, the enriched GO terms were cell adhesion (GO: 0007155, P = 2.26E-19) and negative regulation of apoptotic process (GO: 0043066, P = 3.24E-15), and for cellular component, the enriched GO terms were cytoplasm (GO: 0005737, P = 9.18E-63) and extracellular region (GO: 0005576, P = 2.28E-47) (Figure 2).

To further evaluate the biological significance for the DEGs, we also performed the KEGG pathway enrichment analysis. Hypergeometric test with P value < 0.05 was used as the criteria for pathway detection (Table 3). The most significant pathway in our KEGG analysis was Focal adhesion (P = 5.70E-15). Furthermore, ECM-receptor interaction (P = 1.27E-13) and Cell cycle (P = 4.53E-11) were found to be highly enriched.

**Table 3:** The top 15 enriched KEGG pathway of DEGs

**KEGG ID**	**KEGG Name**	**No.of genes**	**P value**
hsa04510	Focal adhesion	34	5.70E-15
hsa04512	ECM-receptor interaction	22	1.27E-13
hsa04110	Cell cycle	23	4.53E-11
hsa04060	Cytokine-cytokine receptor interaction	28	8.54E-08
hsa04610	Complement and coagulation cascades	14	1.26E-07
hsa05200	Pathways in cancer	31	1.53E-07
hsa04810	Regulation of actin cytoskeleton	22	4.27E-06
hsa04114	Oocyte meiosis	15	1.33E-05
hsa05144	Malaria	10	1.76E-05
hsa04540	Gap junction	13	2.39E-05
hsa04974	Protein digestion and absorption	12	3.59E-05
hsa04380	Osteoclast differentiation	15	5.09E-05
hsa05150	Staphylococcus aureus infection	9	7.59E-05
hsa05322	Systemic lupus erythematosus	12	0.0001029
hsa04142	Lysosome	14	0.000113

### Protein-Protein interaction (PPI) network construction

**Figure 3:**
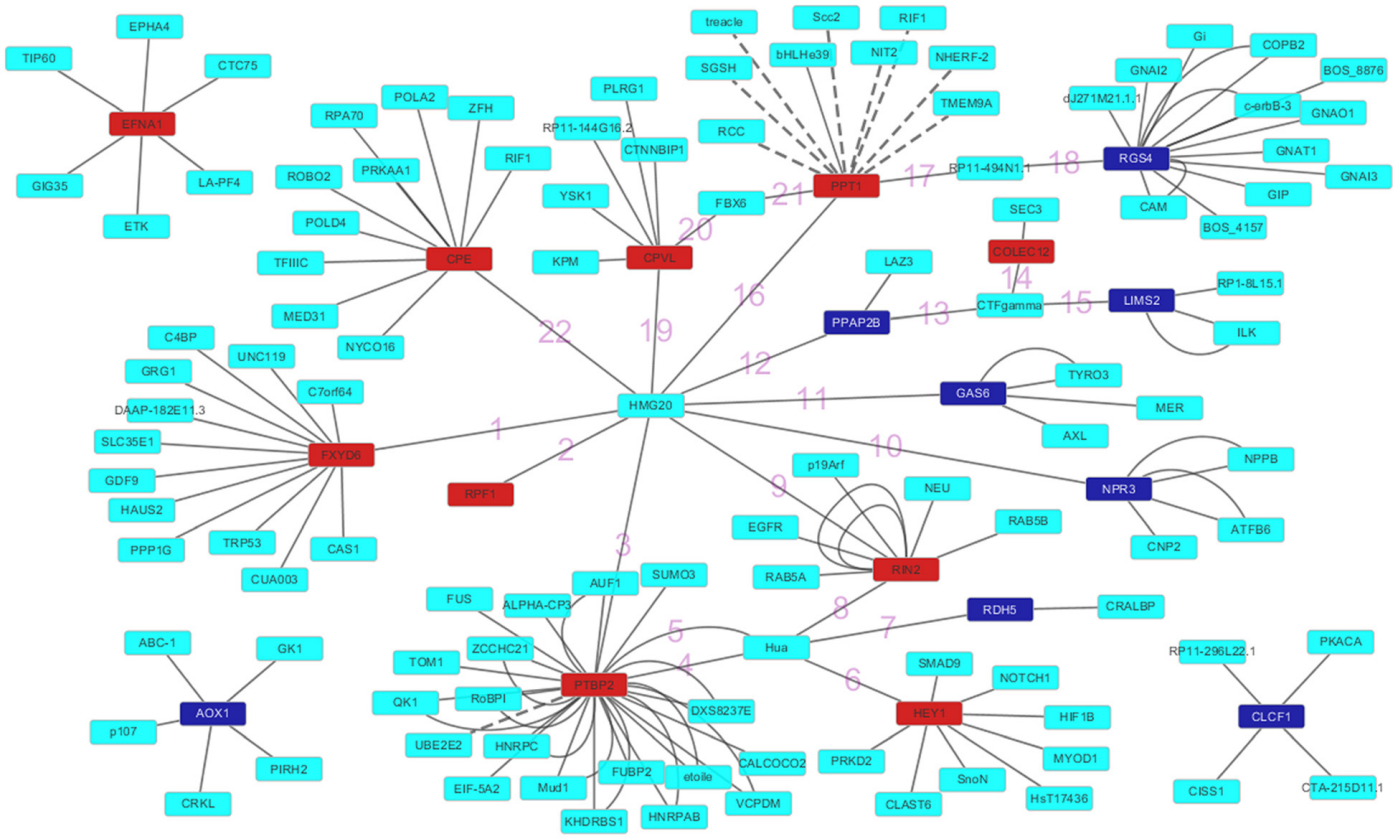
**The constructed protein-protein interaction networks of the top 10 up- and down-regulated DEGs.** The edges numbered mean which connect the top 10 up- and down-regulated DEGs directly or indirectly.

Nodes represent proteins, edges represent interactions between two proteins. The higher the node shape, the greater deagree of connection. The PPI networks we established for the top 10 up-regulated and down-regulated DEGs by Cytoscape software included 129 nodes and 182 edges. The significant hub proteins containing PTBP2 (polypyrimidine tract binding protein 2, Degree = 33), RGS4 (regulator of G-protein signaling 4, Degree = 15) and FXYD6 (FXYD domain containing ion transport regulator 6, Degree = 13) (Figure 3), we also annotated the edges connecting the top 10 up-regulated and down-regulated DEGs directly or indirectly in Additional file 3: Table S2, and numbered in Figure 3.

## Discussion

Osteosarcoma (OS) is an aggressive cancer demonstrating both high metastatic rate and chemotherapeutic resistance. A comprehensive analysis of the mechanism underlying OS development is of crucial importance for management policy. In this paper, we chose a meta-analysis approach that combines differently expressed genes (DEGs) from microarray datasets to highlight genes that were consistently expressed differentially with statistical significance, and performed GO enrichment analysis and KEGG pathway analysis, and construct the protein-protein interaction (PPI) networks.

We performed a meta-analysis using 8 publicly available GEO data sets to identify common biological mechanisms involved in the pathogenesis of OS. OS is a kind of rare tumor, where material from clinical samples is scarce, therefore data from both bone tissues and cell lines were included in our meta-analysis. In total, 979 genes across the studies were consistently expressed differentially in OS (472 up-regulated and 507 down-regulated). The up-regulated gene with the lowest P-value (P = 5.08E-15) was CPE (carboxypeptidase E), which is a carboxypeptidase that cleaves C-terminal amino acid residues and is involved in the biosynthesis of peptide hormones and neurotransmitters, including insulin [24], but at the present the role and association with OS have not yet been reported. In line with previous findings, We found that some genes have been closely related to the development of OS among the top ten up-regulated DEGs, such as HEY1, FXYD6, EFNA1. HEY1, one of target genes of NOTCH1, was reported to be up-regulated in OS from p53 mutant mice, suggesting that activation of Notch signaling contributes to the pathogenesis of OS [25]. Another study also found that HEY1 and other downstream target genes of Notch signaling including HES1, NOTCH1 and NOTCH2, were elevated in canine osteosarcoma by gene expression microarray analysis and reverse transcriptase - quantitative PCR (RT-qPCR) [26]. Olstad OK et al. applied directional tag PCR subtractive hybridization to construct a cDNA library generated from three different human osteosarcoma (OS) target cell lines (OHS, SaOS-2 and KPDXM), and identified FXYD6 was enriched in OS cell lines [27]. EFNA1 was significantly elevated in OS samples by using genome-wide microarrays, and in vitro study on the functional role of EphA2 and EFNA1 showed that EFNA1 ligand binding induced increased tyrosine phosphorylation, receptor degradation and downstream mitogen-activated protein kinase (MAPK) activation [28].

The down-regulated DEGs with the lowest P-value (P = 1.86E-48) was NPR3 (natriuretic peptide receptor 3) that acts as a decoy/clearance receptor functioning to limit the effects of natriuretic peptides. NPR3, which is an important anabolic regulator of endochondral bone growth, is enriched in bone marrow-derived mesenchymal stem cells, and there is no relevant report to OS at present. In our meta-analysis Gas6 was identified to be one of the top ten down-regulated DEGs. In OS cell lines, rhGas6 could activate Axl to protect the tumor cells from apoptosis caused by serum starvation, and promote tumor cells’ migration and invasion in vitro [29].

In order to uncover the biological roles of the DEGs from OS, we performed a GO categories enrichment analysis. We found GO terms for molecular functions significantly enriched in protein binding and calcium ion binding, while for biological processes, the enriched GO terms were cell adhesion and negative regulation of apoptotic process, and for cellular component, the enriched GO terms were cytoplasm and extracellular region. To further evaluate the biological significance for the DEGs, we also performed the KEGG pathway enrichment analysis. Focal adhesion, ECM-receptor interaction and Cell cycle in our KEGG analysis were found to be highly enriched. Many signal transduction pathways involved in OS development were stimulated by bone morphogenetic proteins (BMPs), transforming growth factors (TGFs), Notch family proteins and Wnt family proteins, and components of each of these pathways have been implicated in OS. Interestingly, we noted that the most significant pathway in our KEGG analysis was Focal adhesion. Focal adhesions are associated with cell migration dynamics. However in the human cells focal adhesion would initially appear to be contradictory to their migratory phenotype. It has been proved previously that knockdown of paxillin in highly metastatic OS sub-lines M112 and 132 would inhibit cell migration [30].

Furthermore the results from PPI network analysis of the top 10 up-regulated and down-regulated DEGs indicated the significant hub proteins containing PTBP2, RGS4 and FXYD6. PTBP2, a member of PTB (polypyrimidine tract binding protein) family of RNA-binding proteins which plays a critical role in development through the regulation of post-transcriptional events, is expressed in the nervous system including the brain, the neural retina and the spinal cord and the intermediate mesoderm [31]. PTBP2 regulates the generation of neuronal precursors in the embryonic brain by repressing adult-specific splicing [32], but the function involved in OS development has not been discovered. Our result of PPI suggested that PTBP2 may play an important role in the development.

The present study has some limitations. First, heterogeneity and confounding factors may have distorted the analysis. Clinical samples might be heterogeneous with respect to clinical activity, severity, or gender. Although we conducted global normalization for different data sets, the heterogeneity of various microarray platforms used in different studies can’t remove. Second, there are differences in gene expression between tissues such as bones, cell lines and lung that were not taken into account. However, our meta-analysis integrated data from different studies which may enable us to detect genes that we would otherwise have missed in an analysis. Despite these limitations, our discover have important implications for the molecular mechanisms of OS,but further experimental research is still need to confirm our study.

## Conclusions

In conclusion, by this meta-analysis based on gene expression data of osteosarcoma we have shown the underlying molecular differences between NC tissues and osteosarcoma, including DEGs and their biological function which may contribute to the successful identification of therapeutic targets for osteosarcoma. Further functional studies may provide additional insights into the role of the differentially regulated genes in the pathophysiology of osteosarcoma.

## Competing interests

The authors declare that they have no competing interests.

## Authors’ contributions

YZZ carried out study design, advised on all methodological issues, drafted and critically revised the manuscript. CYB, FY and YYH designed the search strategy, screened data sets for analysis, drafted and critically revised the manuscript. ZY conducted data extraction, transformed into tables, drafted and critically revised the manuscript. CYJ and LDQ screened data sets for analysis, accomplished data analysis, drafted and critically revised the manuscript. All authors approved the final version of this manuscript and agreed to be accountable for all aspects of the work.

## Pre-publication history

The pre-publication history for this paper can be accessed here:

http://www.biomedcentral.com/1471-2350/15/80/prepub

## Supplementary Material

Additional file 1 PRISMA 2009 Checklist. Click here for file

Additional file 2 A list of all DEGs in our meta-analysis. Click here for file

Additional file 3 The annotation of the edges connecting the top 10 up-regulated and down-regulated DEGs directly or indirectly. Click here for file

## References

[B1] Bielack S Carrle D Casali P Osteosarcoma: ESMO clinical recommendations for diagnosis, treatment and follow-up Ann Oncol 2009 15 suppl 4 iv137 iv139 10.1093/annonc/mdp154 19454435

[B2] Grimer RJ Surgical options for children with osteosarcoma Lancet Oncol 2005 15 2 85 92 15683817 10.1016/S1470-2045(05)01734-1

[B3] Akiyama T Dass CR Choong PF Novel therapeutic strategy for osteosarcoma targeting osteoclast differentiation, bone-resorbing activity, and apoptosis pathway Mol Cancer Ther 2008 15 11 3461 3469 19001431 10.1158/1535-7163.MCT-08-0530

[B4] Boehm A Neff J Squire J Bayani J Nelson M Bridge J Cytogenetic findings in 36 osteosarcoma specimens and a review of the literature Fetal Pediatr Pathol 2000 15 5 359 376

[B5] Yen C-C Chen W-M Chen T-H Chen WY-K Chen PC-H Chiou H-J Hung G-Y Wu H-TH Wei C-J Shiau C-Y Identification of chromosomal aberrations associated with disease progression and a novel 3q13. 31 deletion involving LSAMP gene in osteosarcoma Int J Oncol 2009 15 4 775 788 19724913 10.3892/ijo_00000390

[B6] Kresse SH Ohnstad HO Paulsen EB Bjerkehagen B Szuhai K Serra M Schaefer KL Myklebost O Meza‒Zepeda LA LSAMP, a novel candidate tumor suppressor gene in human osteosarcomas, identified by array comparative genomic hybridization Genes Chromosom Cancer 2009 15 8 679 693 19441093 10.1002/gcc.20675

[B7] Paoloni M Davis S Lana S Withrow S Sangiorgi L Picci P Hewitt S Triche T Meltzer P Khanna C Canine tumor cross-species genomics uncovers targets linked to osteosarcoma progression BMC Genomics 2009 15 1 625 20028558 10.1186/1471-2164-10-625 PMC2803201

[B8] Sadikovic B Yoshimoto M Chilton-MacNeill S Thorner P Squire JA Zielenska M Identification of interactive networks of gene expression associated with osteosarcoma oncogenesis by integrated molecular profiling Hum Mol Genet 2009 15 11 1962 1975 19286668 10.1093/hmg/ddp117

[B9] Sadikovic B Yoshimoto M Al-Romaih K Maire G Zielenska M Squire JA In vitro analysis of integrated global high-resolution DNA methylation profiling with genomic imbalance and gene expression in osteosarcoma PLoS One 2008 15 7 e2834 18698372 10.1371/journal.pone.0002834 PMC2515339

[B10] Kuijjer ML Peterse EF van den Akker BE Briaire-de Bruijn IH Serra M Meza-Zepeda LA Myklebost O Hassan AB Hogendoorn PC Cleton-Jansen A-M IR/IGF1R signaling as potential target for treatment of high-grade osteosarcoma BMC Cancer 2013 15 1 245 23688189 10.1186/1471-2407-13-245 PMC3672007

[B11] Kresse SH Rydbeck H Skårn M Namløs HM Barragan-Polania AH Cleton-Jansen A-M Serra M Liestøl K Hogendoorn PC Hovig E Integrative analysis reveals relationships of genetic and epigenetic alterations in osteosarcoma PLoS One 2012 15 11 e48262 23144859 10.1371/journal.pone.0048262 PMC3492335

[B12] Both J Wu T Bras J Schaap GR Baas F Hulsebos TJ Identification of novel candidate oncogenes in chromosome Region 17p11. 2-p12 in human osteosarcoma PLoS One 2012 15 1 e30907 22292074 10.1371/journal.pone.0030907 PMC3266911

[B13] Ying M Liu G Shimada H Ding W May W He Q Adams G Wu L Human osteosarcoma CD49f − CD133&plus; cells: impaired in osteogenic fate while gain of tumorigenicity Oncogene 2013 15 36 4252 4263 23045288 10.1038/onc.2012.438 PMC3947577

[B14] Siddiqui AS Delaney AD Schnerch A Griffith OL Jones SJ Marra MA Sequence biases in large scale gene expression profiling data Nucleic Acids Res 2006 15 12 e83 e83 16840527 10.1093/nar/gkl404 PMC1524917

[B15] Feichtinger J Thallinger GG McFarlane RJ Larcombe LD Microarray meta-analysis: From data to expression to biological relationships Computational Medicine 2012 Springer 59 77

[B16] Ramasamy A Mondry A Holmes CC Altman DG Key issues in conducting a meta-analysis of gene expression microarray datasets PLoS Med 2008 15 9 e184 18767902 10.1371/journal.pmed.0050184 PMC2528050

[B17] Barrett T Wilhite SE Ledoux P Evangelista C Kim IF Tomashevsky M Marshall KA Phillippy KH Sherman PM Holko M NCBI GEO: archive for functional genomics data sets—update Nucleic Acids Res 2013 15 D1 D991 D995 23193258 10.1093/nar/gks1193 PMC3531084

[B18] Tusher VG Tibshirani R Chu G Significance analysis of microarrays applied to the ionizing radiation response Proc Natl Acad Sci U S A 2001 15 9 5116 5121 11309499 10.1073/pnas.091062498 PMC33173

[B19] Tabas-Madrid D Nogales-Cadenas R Pascual-Montano A GeneCodis3: a non-redundant and modular enrichment analysis tool for functional genomics Nucleic Acids Res 2012 15 Web Server issue W478 W483 22573175 10.1093/nar/gks402 PMC3394297

[B20] Giot L Bader JS Brouwer C Chaudhuri A Kuang B Li Y Hao Y Ooi C Godwin B Vitols E A protein interaction map of Drosophila melanogaster Science 2003 15 5651 1727 1736 14605208 10.1126/science.1090289

[B21] Li S Armstrong CM Bertin N Ge H Milstein S Boxem M Vidalain P-O Han J-DJ Chesneau A Hao T A map of the interactome network of the metazoan C. elegans Science 2004 15 5657 540 543 14704431 10.1126/science.1091403 PMC1698949

[B22] Shannon P Markiel A Ozier O Baliga NS Wang JT Ramage D Amin N Schwikowski B Ideker T Cytoscape: a software environment for integrated models of biomolecular interaction networks Genome Res 2003 15 11 2498 2504 14597658 10.1101/gr.1239303 PMC403769

[B23] Murthy SR Pacak K Loh YP Carboxypeptidase E: elevated expression correlated with tumor growth and metastasis in pheochromocytomas and other cancers Cell Mol Neurobiol 2010 15 8 1377 1381 21061162 10.1007/s10571-010-9592-y PMC3057539

[B24] Jeffrey KD Alejandro EU Luciani DS Kalynyak TB Hu X Li H Lin Y Townsend RR Polonsky KS Johnson JD Carboxypeptidase E mediates palmitate-induced β-cell ER stress and apoptosis Proc Natl Acad Sci 2008 15 24 8452 8457 18550819 10.1073/pnas.0711232105 PMC2448857

[B25] Engin F Bertin T Ma O Jiang MM Wang L Sutton RE Donehower LA Lee B Notch signaling contributes to the pathogenesis of human osteosarcomas Hum Mol Genet 2009 15 8 1464 1470 19228774 10.1093/hmg/ddp057 PMC2733809

[B26] Dailey DD Anfinsen KP Pfaff LE Ehrhart E Charles JB Bønsdorff TB Thamm DH Powers BE Jonasdottir TJ Duval DL HES1, a target of Notch signaling, is elevated in canine osteosarcoma, but reduced in the most aggressive tumors BMC Vet Res 2013 15 1 130 23816051 10.1186/1746-6148-9-130 PMC3701487

[B27] Olstad OK Gautvik VT Reppe S Rian E Jemtland R Ohlsson C Bruland O Gautvik KM Molecular heterogeneity in human osteosarcoma demonstrated by enriched mRNAs isolated by directional tag PCR subtraction cloning Anticancer Res 2002 15 3B 2201 2216 12894494

[B28] Fritsche‒Guenther R Noske A Ungethüm U Kuban RJ Schlag PM Tunn PU Karle J Krenn V Dietel M Sers C De novo expression of EphA2 in osteosarcoma modulates activation of the mitogenic signalling pathway Histopathology 2010 15 6 836 850 21166698 10.1111/j.1365-2559.2010.03713.x

[B29] Han J Tian R Yong B Luo C Tan P Shen J Peng T Gas6/Axl mediates tumor cell apoptosis, migration and invasion and predicts the clinical outcome of osteosarcoma patients Biochem Biophys Res Commun 2013 15 3 493 500 23684620 10.1016/j.bbrc.2013.05.019

[B30] Azuma K Tanaka M Uekita T Inoue S Yokota J Ouchi Y Sakai R Tyrosine phosphorylation of paxillin affects the metastatic potential of human osteosarcoma Oncogene 2005 15 30 4754 4764 15870699 10.1038/sj.onc.1208654

[B31] Noiret M Audic Y Hardy S Expression analysis of the polypyrimidine tract binding protein (PTBP1) and its paralogs PTBP2 and PTBP3 during Xenopus tropicalis embryogenesis Int J Dev Biol 2012 15 747 753 23124965 10.1387/ijdb.120017sh

[B32] Licatalosi DD Yano M Fak JJ Mele A Grabinski SE Zhang C Darnell RB Ptbp2 represses adult-specific splicing to regulate the generation of neuronal precursors in the embryonic brain Genes Dev 2012 15 14 1626 1642 22802532 10.1101/gad.191338.112 PMC3404389

